# Analecta Minora

**Published:** 1826-01

**Authors:** 


### XIII.

### ANALECTA MINORA,

OR

### *MINOR PERISCOPE.*

Nihil est aliud magnum quam multa minuta.

1. *Colica Pictonum.* There are various modes of treating this complaint, adopted in different countries. Calomel and opium are often resorted to in England—leeches in France, especially by the school of Broussais, while the peculiar treatment at La Charité is well known. M. Rauque, of Orleans, has published a new method of treatment, which he calls very simple—simple certainly in principle—but most complicated in the means. It is neither more nor less than extensive counter-irritation to the abdomen and loins, by means of a plaster composed of nearly twenty ingredients, the only active articles of which are, tartarized antimony and the lytta. The professor in question considers the disease as a decided neuralgia, seated originally in the solar plexus, and radiating thence to various parts of the abdomen, through the medium of the splanchnic nerves. There is no great objection to this theory, since it is, at least as plausible as the phlogistic hypothesis of the Broussaians. M. Rauque admits, that the neuralgia is often complicated with phlogosis. The mode of treatment, by extensive counter-irritation, answers both indications, and was successful in upwards of 300 cases.

While, on this subject, we may observe that, for some time past, we have been in the habit of using an antimonial plaster instead of the antimonial ointment, wherewith to produce powerful counter-irritation. It is much more easily applied, and we think, much more effectual in the end. It is made by putting about four grains of tartarized antimony to each drachm of Burgundy pitch, and thus the quantity will be regulated by the extent of surface which it is desirable to cover with pustules. Three days is the usual time required. In some persons it will take effect nearly as soon as a blister—and it never requires more than three or four days to produce a close-set crop of pustules, which will keep discharging matter for several days afterwards. We recommend it to our brethren as a most important remedy in affections of the chest and other internal organs—in obstinate head-aches of a neuralgic or inflammatory nature, and in various forms of gastrodynia and dyspepsia.

2. *Disgusting depravation of Taste.* There is now a man in France, aged 30 years, and otherwise healthy in appearance, who exhibits the most revolting perversion in the gratification of his appetite. He tears open graves to devour the entrails of the dead—searches the streets for the putrid pieces of meat which dogs would refuse—and haunts the veterinary hospitals for the *debris* of sores, &c. He has no unnatural appetite, in respect to quantity. When he finds more of these horrible eatables than are sufficient for the moment, he carefully preserves the overplus for another meal. He has been committed to prison for violating the sanctuaries of the dead. When closely interrogated, he evinces some slight aberration of intellect. He is pusillanimous. He avers that he had this depravation of taste from his infancy, and is surprised that he should be found fault with for indulging a *natural* propensity. He has never yet attempted the living body; but confesses that, were he strongly pressed, he would feel little hesitation in seizing on a child, if found sleeping or defenceless! It is high time, we think, to put this man under strict surveillance.—*Archives Generales.*

---

3. *Professional Reclamations.* The dignity of the medical profession is scandalized by some reclamations now publishing in the French metropolis. Thus, in a late sitting of the Royal Academy of Medicine in Paris, M. Baron complained that M. Serres uttered a direct and wilful falsehood, respecting a dissection which M. Baron had made. The latter opened the head of a child, and communicated the results to the Academy. The former, M. Serres, stated the case to another society, and added the dissection of the neck, which he said he had done himself, whereas the neck was not opened by M. Baron himself, till 24 hours after M. Serres had stated the appearances on dissection, in the Philomathic Society! This is dreadful! A committee should have been appointed to examine into this business before such a disgraceful statement was published to the world. What will foreigners think of the heads of the Profession in Paris, after such a scene.

Again:—The veteran Chaussier avers, that he discovered, more than 20 years ago, that albumen was an antidote against the corrosive sublimate and some other metallic solutions, referring for proofs to certain passages in the *Encyclopœdie Methodique* published in 1786. M. Orfila comes forward and avers that this is false, and that no such passages exist in the Encyclopœdia abovementioned! Surely it will not be said "that they manage *these* things better in France!"—*Archives, Mars.*

---

4. *Cicatrisation of large Surfaces.* Mr. Bush, of Frome, has published some cases in a respected cotemporary, shewing the efficacy of a certain mode of treatment in healing large broken surfaces. This treatment consists in touching the edges of the sore daily, with the liquor plumbi acetatis, and then sprinkling the surface with flower, so as to form a scab. Some portions of the scab were daily broken down, so as to permit the discharge of matter. In this way the cure was more quickly accomplished than by the usual surgical dressings with unctuous substances.—*London Medical Journal.*

---

5. *Transfusion of Blood.* Mr. Doubleday has lately performed this operation on a female who, six hours after a tremendous uterine hæmorrhage, was sinking from inanition, in despite of brandy, laudanum, ammonia, and other stimuli. The first injection of a few ounces of blood from the husband's arm, lowered the pulse from 140 to 104, and soon afterwards to 90, while it became proportionally expanded. The whole case, which has been minutely detailed and laid before the Medical Society of London, has convinced us that the operation was not only innocuous in this instance, but, in all human probability, was the means of saving the woman's life. In the protracted discussions which took place in the Society upon this case, various objections were raised, some hypothetical, some futile, and many of them both frivolous and vexatious. An unbiassed and candid attention to the details has led us to the foregoing conclusion—and we think Mr. Doubleday entitled to the highest praise for his prompt and manly exertions in behalf of a dying fellow creature. We advise him to publish the case in detail, and we have no doubt that the public generally will come to the same conclusion which we have come to. N.B. The case has been since published.

6. *Oil of Turpentine in Purpura.* Dr. Magee, of Dublin, in the relation of a case of purpura hæmorrhagica, treated with oil of turpentine, observes, that he has found the following to be the best mode of administering this nauseous, but valuable medicine. To an adult he gives half an ounce, mixed with an equal quantity of castor oil, and in conjunction with a little cinnamon or peppermint water. The dose to children will be easily graduated from the above scale. He avers that, in this form, it rarely is rejected by the stomach, and will often remain when opiate draughts are thrown off.

---

7. *Tetanus cured.* Dr. Alexander, of Prince of Wales' Island, lately brought to a successful issue, an unequivocal case of idiopathic tetanus, by means of copious and repeated general bleeding—numerous leeches to the abdomen—active purgation—and mercurial impregnation of the system.—*Ed. Journal.*

---

8. *Forced Injections for retention of Urine.* It will appear curious, if not incongruous, that, when a bladder is distended with urine, and cannot disgorge itself in consequence of a stricture in the urethra, the patient may be relieved by *forcing* some more fluid into the bladder. It is to be remembered, however, that the cause of the retention, in such cases, is not the fulness of the bladder, but the obstruction in the passage. This obstruction being overcome by a stream forced inwards, the bladder may then be able to relieve itself. Such, at least, are the assertions of a French surgeon, M. Amusat, (before the Academy of Surgery in the French metropolis,) who appeals to facts in support of his assertions. M. Amusat passes a flexible tube as far as the obstruction, and then, by means of an elastic gum-bottle, gently forces a small quantity of fluid through the stricture. The consequence is, (and a number of trials have been made) that the urine flows out again, and the retention is relieved. The experiment involves no danger, and certainly may be fairly had recourse to, previously to the operation of puncturing the bladder, or using much force for the introduction of a bougie.

---

9. *National Compliments.* The Journal *Complémentaire,* for April last, pays the British Medical Profession a flattering compliment, while speaking of a little work by Dr. Fordyce, translated into French, on a new plan of taking down cases. "The English Physicians," says the reviewer, "should read this little work with attention, in order that they may no longer inundate their books and journals with *cases,* in which not the slightest trace of talent for observation is ever to be seen."

We must acknowledge that *cases* in this country are very often published in a slovenly and uncouth manner, which, however, need not have drawn down such a sweeping censure from our Gallic cotemporary. It is one thing to *observe* well, and another to *act* judiciously. The French observe with minute attention—so does the bird while eyeing the snake, but makes no effort to parry the danger which it sees.—*Verbum sapienti.*

While on the subject of national compliments, let us congratulate our respected cotemporary of Philadelphia, (the Philadelphia Journal of the Medical and Physical Sciences) on a return of amicable feeling towards us of the old world, as evinced by wiping away the motto which it had long adopted,* and which was well calculated to keep up feelings of enmity between the two nations. Such emblems of hostility may suit political controversies,

* "Who reads an American book, &c." ?—From the Edinburgh Review.

but should never be permitted to disgrace the fair page of science—and especially medical science, the child of no particular country, but the benefactor of all. We give ourselves some credit for having been instrumental in the removal of the irritating motto. We beseeched the editors to efface it some time ago.

---

10. *Leeches.* In our last number we communicated the means by which the Neapolitans induce leeches to fix on any particular spot—namely, by touching the part with the point of a quill recently taken from a pigeon's wing. We have tried the experiment, in several instances, since our last, and always found it succeed. We have now to notice another mode of whetting the appetites of these little animals—which is neither more nor less than by putting them into some porter for a few minutes, when they will be found to bite very greedily. By an additional dip in this favourite beverage, they will be induced to renew their labours, after disgorging the contents of their first cargo.

---

11. *Gastrotomy.* It appears that Dr. Ben. Day, of Maryland, has succeeded in removing an extra-uterine fœtus from the abdomen of a woman, who was *in extremis* before the operation. The incision was made on the left of the linea alba, and was not above six inches in extent. Through this opening the fœtus was extracted piecemeal, and the woman perfectly recovered.—*Med. Recorder, July*, 1825.

---

12. *Extra and Super-fœtation.* Dr. Trezevant (of South Carolina) was called upon to examine a negress who died suddenly. A large quantity of blood was found among the intestines, and also a fœtus of perfect form, nine inches long. It had been contained in the right ovarium or the fallopian tube, as neither of them could be discovered, and the sac in which the fœtus was found, appeared to occupy their place. The fœtus evinced no symptoms of decay. "Upon removing the membranes from their attachment, for the purpose of examining the placenta, I was struck with the appearance of a large vesicle, about the size of a walnut, on the outer surface of the membranes of the fœtus. Upon opening this, a small fœtus, of about from six to eight weeks old, was discovered. This fœtus was also perfect. It had its investing membranes, a jelly-like cord, and a placenta, which it appeared to have commenced forming on the external portion of the investing membranes of the larger one." This smaller fœtus had the appearance of being recently alive, its body being full and plump, all the features expanded and perfect, "and the red blood evident in its vascular system." The preparation is in the Doctor's possession, the dissection being witnessed by several other practitioners. This fact would seem strongly to favour the doctrine of super-fœtation, as it can hardly be supposed that both the above were the result of one conception, and that the difference of development was owing to the death of one at an early period.—*Med. Recorder, July*, 1825.

---

13. *Tetanus cured.* Dr. Reese, of Baltimore, has communicated the particulars of a case of traumatic tetanus, in which the disease was controlled by a caustic issue applied to the spine, and of which we shall take some short notice in this place.

A young lad, of 15 years of age, received a quantity of buck-shot in his back, from a loaded gun. Two of the shot entered the spine, followed by total paralysis of the parts below. Next day, some re-action having taken place, it was necessary to bleed and to employ the catheter, this last operation being necessary twice or thrice a day, in consequence of the pain occasioned by distention of the bladder. The most drastic purgatives produced little or no effect. In seven days, symptoms of tetanus came on, in the form of opisthotonos, which threatened the life of the patient. Tincture of opium, in drachm doses, was given every half hour, during the night, and on the next day, it was determined, in consultation with Professor Pattison, to try the effect of the extensive application of the caustic potass along the whole length of the spine. At the suggestion of Professor Pattison, the Prussic acid was also exhibited. At this time the patient's body was contracted backwards to a semicircle—his feet distorted, the toes being drawn inward to an almost incredible degree—while he was suffering exquisite torture from the contraction of the muscles, causing him to shriek most piteously. In four hours after the application of the caustic to the whole of the spine, there was a mitigation of the spasms. He fell into a sleep, which continued six hours, and awoke free from tetanic symptoms, and with only the paralysis of the lower extremities and bladder remaining. In this case there was perfect sensation where the power of motion was entirely lost. When the account was published, the boy had so far recovered, as to be able to walk with a stick.

The Prussic acid being given in this instance, some doubt may exist as to the efficacy of the caustic. But the dose was only half a drop every hour, so that only two drops could have been given previously to the relief obtained. The powerful counter-irritation of a caustic of such extent as the above, was more likely to be the remedial agent (if it was not a natural cessation of the spasm) than such a trifling quantity of the Prussic acid.—*Med. Recorder, July.*

---

14. *Internal Abscess.* The error of diagnosis, in the following case, will point out the utility and importance of examining the chest by means of auscultation and percussion.

On the 21st July, 1823, Dr. Reese, of Baltimore, was called to a lady, whom he found labouring under extreme debility, from a protracted affection which had been treated as dyspepsia, originating in chronic hepatitis. On examination of the symptoms, and a review of the history, Dr. R. gave it as his opinion that an abscess, probably of the liver, existed. Dr. R. does not tell us what the symptoms were which authorised this diagnosis as to the *seat* of the abscess. There were rigors and night-sweats; but these are common to abscesses every where. Tonics were given till the 29th of July, and had the effect of improving the appetite and general strength. On the night of the last date, a sudden discharge of pus, *sursum ac deorsum*, to the amount of nearly four pints, took place, attended by syncope and other alarming symptoms. Stimuli were given—a blister was applied to the epigastrium—and the denuded surface was dressed with mercurial ointment. This last induced a gentle ptyalism in twelve hours, with much relief. The pus continued to be discharged through the bowels for four or five days, but the patient rallied greatly in this time. Our author stiil continued to think the matter came from the liver. Phlegmasia dolens now supervened, and harassed the patient for three weeks; her health and strength recruiting nevertheless. She so far recovered as to go out riding, and even using the cold bath. She now got a severe cold, was seized with pneumonia, was bled—and died. On dissection, no trace of abscess was found beneath the diaphragm. Some pus was contained in the stomach, similar to what she had formerly passed. "On opening the thorax, we perceived, on the left side of the mediastinum, a collection of fœtid purulent matter, perhaps about a pint, precisely similar to that in the stomach, and to that which passed off by the intestines 32 days previously. The left lung, at its inferior point, shewed marks of lesion of its substance and chronic derangement. We were still at a loss to know how the pus escaped into the stomach; but, on passing my finger up the œsophagus, I discovered the point at which it had opened into this canal about an inch and a half from the cardiac orifice of the stomach."

Now, we may be permitted to remark on this case, that, had the chest been examined by auscultation, or even percussion, the existence of this depôt of purulent matter would have been detected without the slightest difficulty, and thus the erroneous *prognosis* would have been saved; for our author left off his attendance and pronounced his patient convalescent, with a large depôt of purulent matter in the chest! But this is not all. Had the disease been investigated in the proper manner, the patient would not have been recommended to ride out and use the cold bath; but, on the contrary, quietude, seclusion, and gentle tonics, might have conducted to a safe termination; the abscess having fortunately found for itself an opening into the line of the primæ viæ. Will any one say, after this, that auscultation is a useless piece of refinement?—*Med. Recorder, July*, 1825.

---

15. *Death from an Ascarid. Lumbricoid.* It has been denied by certain herminthologists, and especially by Bresmer and Rudolphi, that the worm in question is capable of piercing the intestinal tube, since it is destitute of an apparatus for that purpose. The following case, by M. Fontanelles, as observed in the Great Hospital of Milan, is published in our respected cotemporary, the Revue Medicale, as contradictory of the opinions of the abovementioned physicians.

*Case.* J. Montovani, aged 13, of Milan, was brought to the Hospital on the 25th April, 1824. He had been affected, for ten or twelve days, with convulsive movements of the right arm and leg, without any loss of sensibility in the parts. He appeared to have been previously in health. Soon after entering the hospital he had a short paroxysm of epilepsy, and he was in a stupid state, which prevented any more history of the case being procured. At first he was thought by Acerbi, the physician, to be labouring under St. Vitus's dance; but, on the second visit, (same day) he recognized a case of severe eclampsia. Three grains of tartar emetic, dissolved in four ounces of water, with tamarind decoction, to be taken in the 24 hours. 26th. No vomiting, no stools since yesterday. Six grains of antimony for the next 24 hours. This dose was, by mistake, taken all at once, and only produced one motion. 27th. The spasms of the arm and leg were the same. He had now smart fever, the heat of the skin being increased, the eyes sparkling and bloodshot, perspiration copious, breathing short and laborious. Venesection to 10 ounces—four grains of antimony. 28th The blood shewed no signs of inflammation—no vomiting—some stools. He had several epileptic seizures, and the convulsive movements continued. The patient's intellectual functions were completely lethargic. Eighteen leeches to the temples. Two grains of calomel and two of gamboge every three hours. 29th. Had two motions. He is worse in all respects. Died in the night.

*Dissection.* Dura mater red and much injected. Some serosity under the arachnoid, together with some coagulable lymph. Substance of brain and spinal marrow sound. The lungs were gorged, but there was no other morbid appearance in the thorax. In the stomach were found several lumbrici, with some degree of vitality remaining—no sign of inflammation in the lining membrane of the stomach. The mucous coat of the duodenum was very much injected, and appeared actually inflamed. A lumbricus, six inches in length, was found to have penetrated the ductus communis choledochus, reaching as high as the junction of the ductus cysticus and d. hepaticus. The ductus communis was rent by the worm, the head of which came out through a hole in the ductus hepaticus. The gall-bladder was very much distended with viscid bile. Some lumbrici were found in the inferior part of the duodenum, and in the whole intestinal canal there were twenty, in number, of the same animals. There was no other organic lesion than that abovementioned.

This is a very curious case. There can be little doubt that the sympathetic disturbance in the nervous system, which went the length of destroying life, was the consequence of the irritation produced by the worm penetrating the biliary ducts. The inflammation in the mucous membrane of the duodenum was the effect of the irritation, and can hardly be looked upon as the cause of death.

---

16. *Electricity.* Since the discovery of electricity, the most distinguished philosophers have concurred in regarding it as the principal agent in some of the most important phenomena of animal life. Their opinion, however, for a long time seemed to be supported rather by analogy than by direct evidence. Comparatively but a few months have elapsed since two Swiss physiologists, Prevost and Damas, proved that muscular contractions, in whatever manner excited, whether mechanically or chemically, are invariably accompanied by a development of electricity. It still remained to be decided whether this electricity be necessarily present as the essential cause, or merely as an accidentally associated phœnomenon. The following experiments appear to carry us a step farther towards the decision of the question.

Dr. Edwards has investigated the effects produced by touching a nerve in a manner which had been but little attended to. It consists in passing a solid body along a nerve, in the same manner in which we pass a magnet along a bar of steel which we wish to magnetize.

He conducts the experiment in the following manner:—He lays bare the sciatic nerves of a frog, in that part of their course in which they are situated on the sacrum, leaving unimpaired their connexion with the spinal marrow and with the muscles to which they are directed. He removes the skin from the posterior extremities, that the movements of the muscular fibres may be visible, and intercepts volition by dividing the spinal marrow just below the head. He then places under the nerves a slip of oiled silk by which they are raised and supported on a level with the bone. If a metallic rod be now drawn lightly along the denuded nerve in the mode above-mentioned, muscular contractions will be excited. This effect is produced, whatever be the metal employed. It is not even necessary that the rod should be metallic. Horn, glass, ivory, or any other solid body, will answer the purpose, but their influence is by no means the same. Though Dr. Edwards clearly ascertained this fact, continual variations in the irritability of the animal precluded the possibility of establishing a scale. The Doctor then substituted for the oiled silk, which is a very complete non-conductor of electricity, a slip of muscle perfectly similar as to form and size, but which it will be remarked is an excellent conductor. A repetition of the contact now no longer caused contractions, or, at most, they were extremely feeble. In the first experiment, the electricity developed by the contact of the nerve is retained, and its influence is concentrated on the nerve itself. In the second case, the electricity is abstracted. If the presence of electricity were merely adventitious, Dr. Edwards thinks that the same mechanical excitation ought, in both cases, to produce the same effect, but the difference is decided. He, therefore, concludes that electricity is essential to muscular contraction.

